# Seroprevalence and Cross-reactivity of Human Polyomavirus 9

**DOI:** 10.3201/eid1808.111625

**Published:** 2012-08

**Authors:** Jérôme T.J. Nicol, Antoine Touzé, Rémy Robinot, Francoise Arnold, Elisa Mazzoni, Mauro Tognon, Pierre Coursaget

**Affiliations:** Institut National de la Santé et de la Recherche Médicale, Tours, France (J.T.J. Nicol, A. Touzé, R. Robinot, F. Arnold, P. Coursaget);; Université François Rabelais, Tours (J.T.J. Nicol, A. Touzé, R. Robinot, F. Arnold, P. Coursaget);; and School of Medicine, University of Ferrara, Ferrara, Italy (E. Mazzoni, M. Tognon)

**Keywords:** HPyV9, human polyomavirus 9, LPyV, MCPyV, seroprevalence, viruses, polyomavirus, baculovirus, cross-reactivity, simian lymphotropic virus

## Abstract

Many humans have antibodies against simian lymphotropic polyomavirus (LPyV), but its DNA has not been found in humans. Identification of human polyomavirus 9 (HPyV9) led us to compare the seroprevalence and cross-reactivity of LPyV and HpyV9. Results could indicate that humans who have antibodies against LPyV are infected by HPyV9.

To date, 9 human polyomaviruses (HPyVs) have been identified: BK polyomavirus, JC polyomavirus, Karolinska Institute polyomavirus, Washington University polyomavirus, human polyomavirus 6 and 7, Trichodysplasia spinulosa-associated polyomavirus, Merkel cell polyomavirus (MCPyV), and human polyomavirus 9 (HPyV9), which was identified in 2010 in human blood and skin samples (*1**,**2*). Serologic studies have shown that most adults have been exposed to HPyVs, and cross-reactivity studies have shown antigenic similarities between simian virus 40 and BK polyomavirus and, to a lesser extent, between simian virus 40 and JC polyomavirus (*3**–**5*). Interpretation of phylogenetic analysis of viral protein 1 (VP1) sequences predicts that cross-reactivity might also occur between Trichodysplasia spinulosa-associated polyomavirus and Bornean orangutan PyV, between MCPyV and chimpanzee polyomaviruses, and between HPyV9 and simian lymphotropic polyomavirus (LPyV).

Serologic survey results have shown that 15.0%–30.0% of humans have antibodies against LPyV, suggesting that the human population has been exposed to an antigenically related PyV (*4**–**6*). However, because LPyV DNA sequences have not been reported among humans (*7**,**8*), LPyV has thus far been considered to be a virus with a narrow host range, limited to nonhuman primates. The 2010 identification of HPyV9 (*1**,**2*), a virus phylogenetically related to LPyV (84.0% of identity), led us to investigate the seroprevalence of this newly discovered polyomavirus and to evaluate the existence of cross-reactivity that might explain the LPyV seroprevalence in humans.

## The Study

We investigated the seroprevalence of HPyV9, MCPyV, and LPyV in children 1–14 years of age and adults18–85 years of age in the Ferrara region of Italy during December 2010–September 2011, and attempted to determine whether cross-reactivity between LPyV and HPyV9 might explain the latter’s seroprevalence reported among humans. Serum samples from 139 children (63 boys, 76 girls) and 186 adults (82 men, 104 women) were analyzed for HPyV9, LPyV, and MCPyV antibodies. The serum samples from healthy adult blood donors aged 18–65 years were obtained from the University Hospital of Ferrara Blood Center, and samples from children and from adults 66–85 years of age were obtained from the Clinical Analysis Laboratory, University Hospital of Ferrara, by using a protocol approved by the local county ethics committee at University of Ferrara. Consent from participants was not requested for polyomavirus testing; the identity of the sources was removed from the samples, and they were analyzed anonymously. To detect antibodies against HPyV9, we set up a virus-like particle (VLP)–based ELISA similar to the assay we developed for MCPyV (*9*). We obtained the HPyV9 VP1 coding sequence by total synthesis with a codon usage adapted for expression in *Spodoptera frugiperda* cells (Genscript, Piscataway, NJ, USA) (GenBank accession no. HQ696595). We used the Bac-to-Bac Baculovirus Expression System (Invitrogen, Fisher Scientific, Illkirch, France) to generate recombinant baculoviruses. HiFive cells maintained in Grace medium (Invitrogen) were infected with the different recombinant baculoviruses for the production of the different VLPs. VLPs were purified, and the presence of VLPs was determined by electron microscopy (*9*) (Technical Appendix Figure 1).

ELISAs were performed (*9*) in wells coated with 100 ng of VLPs. We determined concentration of HPyV9, LPyV, and MCPyV VLPs by using the Qubit Protein Assay Kit (Invitrogen). We used human serum samples diluted 1:100 and peroxidase-conjugated goat anti-human IgG (Clinisciences, Nanterre, France) diluted 1:20,000 to detect binding of human IgG. The cutoff values for all assays were set at 0.2, as determined previously for MCPyV and LPyV (*9*).

**Figure 1:**
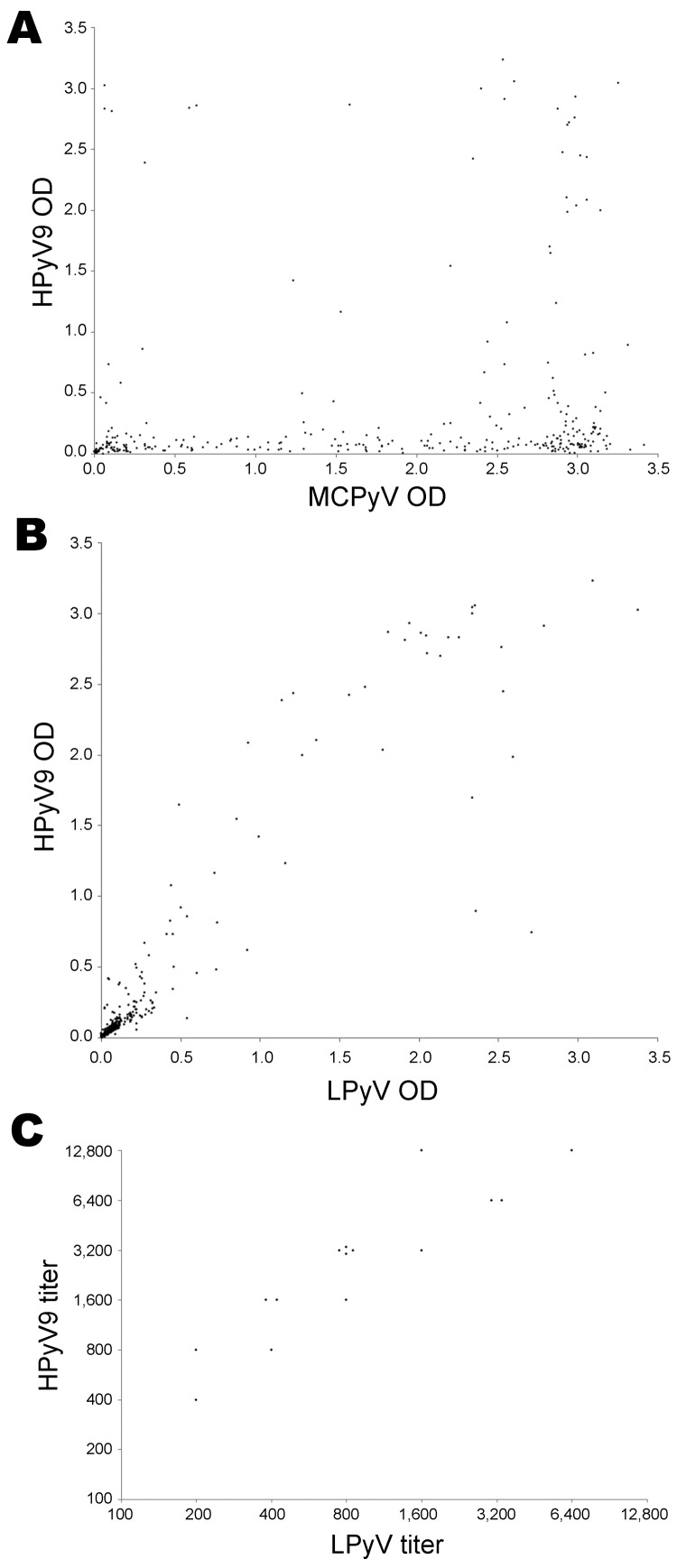
Cross-reactivity between human polyomavirus 9 (HPyV9), simian lymphotropic polyomavirus (LPyV), and Merkel cell polyomavirus (MCPyV) virus–like particles (VLPs). Correlation between A) seroreactivity of 325 serum samples from children and adults in Italy against HPyV9 VLPs and B) MCPyV VLPs and HPyV9 VLPs was analyzed by ELISA. Each point represents 1 serum sample. Correlation coefficients (Spearman ρ) were determined by using XLStat software (Addinsoft, Paris, France). Correlation coefficient was 0.842 in panel B and 0.139 in panel A. C) Titers of 15 serum samples from adults against HPyV9 VLPs and LPyV VLPs. Each point represents 1 serum sample. OD, optical density.

In all, 81 (24.9%) of the 325 investigated study participants were seropositive for HPyV9, 82 (25.2%) were positive for LPyV, and 252 (77.5%) were seropositive for MCPyV. Seventy-six HPyV9-positive participants were also LPyV positive, whereas the ELISA optical density was found to be close to the cutoff value for the remaining 11 discordant cases. We performed proportional analysis between groups, correlation analysis between HPyV9 and LPyV reactivity and between HPyV9 and MCPyV reactivity by using the χ^2^ test and the nonparametric Spearman test, respectively, using XLStat software (Addinsoft, Paris, France). The antibody reactivity of the 325 samples analyzed showed no correlation between HPyV9 and MCPyV ELISA results (ρ = 0.14, p = 0.0001; Figure 1, panel A) but a strong correlation between HPyV9 and LPyV ELISA results (ρ = 0.84, p = 0.001; Figure 1, panel B). In addition, antibody titers against HPyV9 and LPyV were determined for a subset of 15 human samples. As shown in Figure 1, panel C, the antibody titer against HPyV9 was always higher than the titer against LPyV (mean geometric antibody titer 2,660 against HPyV9 and 877 against LPyV).

To assess the degree of antigenic cross-reactivity between HPyV9 and LPyV, we performed competition assays by preincubation (1 h at 37°C) of the 15 anti-HPyV9–positive serum samples (diluted 1:100) by using 2 µg of HPyV9, LPyV, and MCPyV VLPs. In HPyV9 ELISA, the reactivity declined dramatically to undetectable levels after preincubation by HPyV9 VLPs but not by LPyV and MCPyV VLPs (Table). Similarly, the serum reactivity in LPyV ELISA declined to undetectable levels (or to low levels in 3 cases) after preincubation with both HPyV9 and LPyV but not with MCPyV VLPs. Dose-ranging competition experiments were performed on serum samples from 2 persons (Technical Appendix Figure 2), confirming the specificity of the inhibition. These results indicate that the HPyV9 reactivity detected in human serum samples was caused by HPyV9 infection and not by infection with the closely related LPyV. The findings also indicate that detection of LPyV antibodies in human serum samples should be attributed to cross-reacting HPyV9 antibodies.

**Table:** Cross-competition between HPyV9, LPyV, and MCPyV VP1 in serum samples reactive against HPyV9 and LPyV VP1 virus-like particles*

Serum sample no.	HPyV9 reactivity (OD) after preincubation with					LPyV reactivity (OD) after preincubation with			
	Control	HPyV9	LPyV	MCPyV		Control	HPyV9	LPyV	MCPyV
H1	0.988	0.054	0.737	0.943		0.848	0.049	0.036	0.841
H2	1.083	0.128	0.737	1.018		1.007	0.128	0.104	0.995
H3	1.154	0.100	0.921	1.128		1.080	0.073	0.061	1.159
H4	2.147	0.164	1.308	1.869		1.680	0.126	0.100	1.629
H5	2.256	0.195	2.023	2.152		2.525	0.349	0.153	2.671
H6	1.610	0.127	0.846	1.544		2.068	0.099	0.095	2.179
H7	1.223	0.088	0.577	1.456		1.816	0.069	0.062	1.828
H8	1.136	0.017	0.629	0.935		0.599	0.015	0.162	0.545
H9	0.732	0.055	0.437	0.648		0.441	0.119	0.075	0.448
H10	1.216	0.036	0.683	1.057		0.771	0.045	0.032	0.659
H11	1.489	0.070	1.114	1.303		0.801	0.203	0.070	0.522
H12	1.406	0.016	0.776	1.265		0.885	0.041	0.032	0.858
H13	1.682	0.042	0.944	1.199		0.853	0.168	0.065	0.547
H14	2.424	0.061	1.566	2.145		1.536	0.064	0.058	1.516
H15	2.843	0.067	2.109	2.611		2.339	0.075	0.318	2.205

*HPyV9, human polyomavirus 9; LPyV, simian lymphotropic polyomavirus; MCPyV, Merkel cell polyomavirus; VP1, viral protein 1; OD, optical density. Fifteen serum samples from blood donors reactive against HPyV9 and LPyV VP1 virus-like particles were preincubated with HPyV9, LPyV, or MCPyV VLPs.

**Figure 2:**
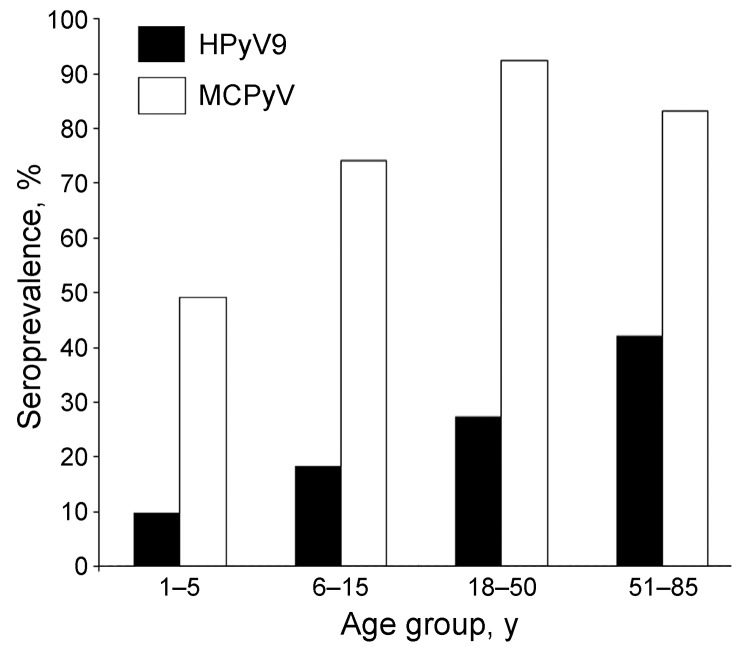
Human polyomavirus 9 (HPyV9) and Merkel cell polyomavirus (MCPyV) seroprevalence in children and adults in Italy.

HPyV9 infection was confirmed in 27.5% of adults18–50 years of age and in 42.4% of adults 51–85 years of age (Figure 2). MCPyV infection was confirmed in 82.5% and 83.3%, respectively, of the same population groups. Among children 1–7 years of age, few HPyV9-positive subjects (10.1%) were detected compared with MCPyV-positive subjects (49.3%). HPyV9 and MCPyV antibodies were detected in 18.6% and 74.3% of children 7–14 years of age, respectively. No difference in HPyV9 seroprevalence was observed among children according to sex (14.3% vs. 14.5%, p = 0.98). Among adults, HPyV9 seroprevalence was higher among men than women (47.6% vs. 21.1%, p = 0.007). No difference in MCPyV antibodies according to sex was observed among children (69.8% vs. 55.3%, p = 0.39) or adults (86.6% vs. 91.3%, p = 0.80).

## Conclusions

The HPyV9 antibodies detected in humans did not cross-react with MCPyV VLPs but cross-reacted strongly with LPyV VLPs in agreement with the recently published findings by Trusch et al., who used VP1 capsomers (*10*). Moreover, inhibition analysis suggested that LPyV does not infect humans or is limited in scope and does not circulate widely among humans.

Our finding that 61 (32.8%) of 186 adults were seropositive for HPyV9 supports the view that this virus is commonly circulating and infects humans throughout life. However, the HPyV9 seroprevalence was low compared with that observed for other human polyomaviruses (*3**,**5**,**11**–**14*), with the exception of that for human polyomavirus 7 (*13*).

Primary exposure for other HPyVs often occurs in early childhood, as found for MCPyV in this study and others (*5**,**12*). The detection of antibodies in 10.1% of children1–7 years of age and the increasing seroprevalence related to increasing age among adults suggests that infection by HPyV9 occurs at all ages. It is not surprising that HPyV9 seroprevalence is lower than the seroprevalence observed for other polyomaviruses because HPyV9 DNA has been detected in only a few persons (*1**,**2*), compared with the high frequency reported for other skin polyomaviruses (*13**,**15*). In conclusion, HPyV9 circulates widely in humans but not as commonly as other polyomaviruses. Further studies are needed to identify the clinical role of this polyomavirus.

Technical Appendix Electron micrographs of viral structures obtained by expression of viral protein 1 genes from human polyomavirus 9 (HPyV9), Merkel cell polyomavirus (MCPyV), and simian lymphotropic polyomavirus (LPyV) in insect cells using recombinant baculoviruses, and seroreactivity of 2 human serum samples to HPyV9 virus–like particles after preincubation with increasing amounts of HPyV9-, LPyV-, and MCPyV-virus–like particles.
