# Supplementary material for: Seroprevalence and Cross-reactivity of Human Polyomavirus 9
Source: Emerg Infect Dis. 2012 Aug;18(8):1329–32. doi: 10.3201/eid1808.111625 (PMC3414022; doi:10.3201/eid1808.111625)
Supplement: Technical Appendix — Electron micrographs of viral structures obtained by expression of viral protein 1 genes from human polyomavirus 9 (HPyV9), Merkel cell polyomavirus (MCPyV), and simian lymphotropic polyomavirus (LPyV) in insect cells using recombinant baculoviruses, and seroreactivity of 2 human serum samples to HPyV9 virus–like particles after preincubation with increasing amounts of HPyV9-, LPyV-, and MCPyV-virus–like particles. [file 11-1625_Techapp-s1.pdf]

Article DOI: http://dx.doi.org/10.3201/eid1808.111625

Seroprevalence and Cross-reactivity of Human Polyomavirus 9

Technical Appendix

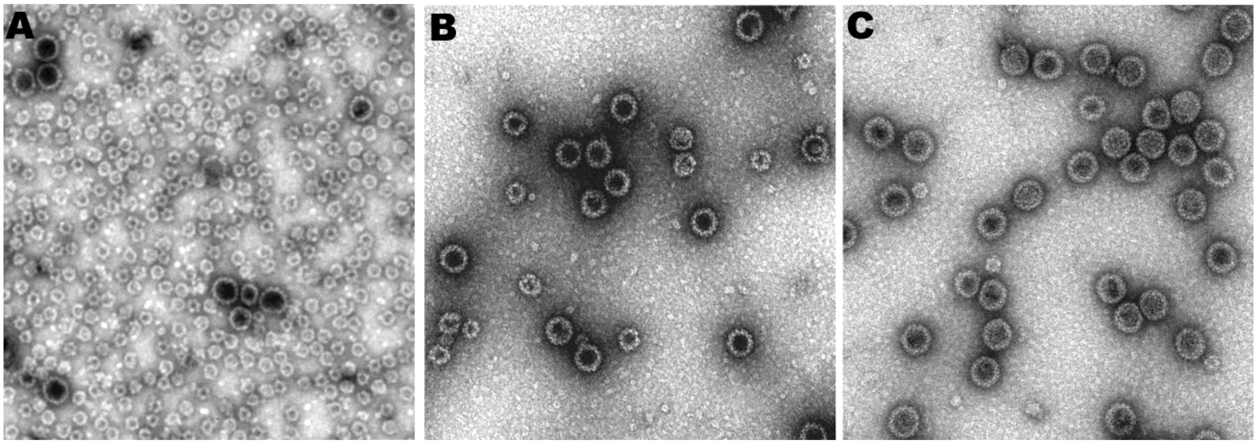

Technical Appendix Figure 1. Electron micrographs of viral structures obtained by expression of viral protein 1 (VP1) genes from human polyomavirus 9 (HPyV9), Merkel cell polyomavirus (MCPyV), and simian lymphotropic polyomavirus (LPyV) in insect cells using recombinant baculoviruses. The preparations were applied to carbon-coated grids, negatively stained with 1.5% uranyl acetate, and observed at 50,000 nominal magnification with a JEOL 1011 electron microscope (JEOL (Europe) SAS, Crossy-sur-Seine, France). Scale bars = 100 nm. In all preparations, 45-nm particles were observed, but 24-nm particles that might correspond to T = 1 structures were present at high concentrations in the HPyV9 preparations.

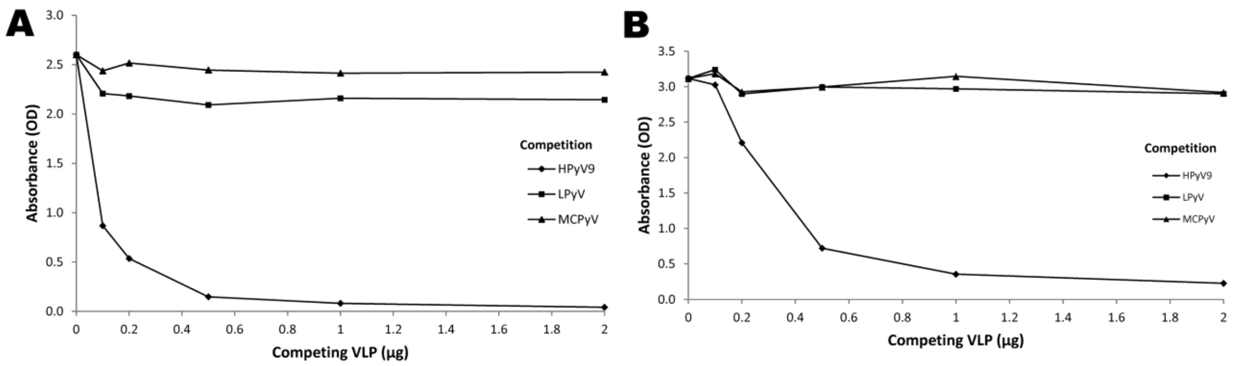

Technical Appendix Figure 2. Seroreactivity of 2 human serum samples (A, B) to human polyomavirus 9 (HPyV9) virus–like particles after preincubation with increasing amounts of HPyV9-, LPyV-, and MCPyV-virus–like particles.
